# A global database of plant production and carbon exchange from global change manipulative experiments

**DOI:** 10.1038/s41597-020-00661-5

**Published:** 2020-10-02

**Authors:** Jian Song, Jingyi Ru, Mengmei Zheng, Haidao Wang, Yongge Fan, Xiaojing Yue, Kejia Yu, Zhenxing Zhou, Pengshuai Shao, Hongyan Han, Lingjie Lei, Qian Zhang, Xiaoming Li, Fanglong Su, Kesheng Zhang, Shiqiang Wan

**Affiliations:** 1 School of Life Science, Institute of Life Science and Green Development, Hebei University, Baoding, Hebei 071002 China; 2 International Joint Research Laboratory for Global Change Ecology, School of Life Sciences, Henan University, Kaifeng, Henan 475004 China; 3 Shandong Key Laboratory of Eco-Environmental Science for the Yellow River Delta, Binzhou University, Binzhou, Shandong 256603 China; 4 Sino-French Institute for Earth System Science, College of Urban and Environmental Sciences, Peking University, Beijing, 100871 China; 5 Luoyang Institute of Science and Technology, Luoyang, Henan 471023 China

**Keywords:** Ecosystem ecology, Climate-change ecology

## Abstract

Numerous ecosystem manipulative experiments have been conducted since 1970/80 s to elucidate responses of terrestrial carbon cycling to the changing atmospheric composition (CO_2_ enrichment and nitrogen deposition) and climate (warming and changing precipitation regimes), which is crucial for model projection and mitigation of future global change effects. Here, we extract data from 2,242 publications that report global change manipulative experiments and build a comprehensive global database with 5,213 pairs of samples for plant production (productivity, biomass, and litter mass) and ecosystem carbon exchange (gross and net ecosystem productivity as well as ecosystem and soil respiration). Information on climate characteristics and vegetation types of experimental sites as well as experimental facilities and manipulation magnitudes subjected to manipulative experiments are also included in this database. This global database can facilitate the estimation of response and sensitivity of key terrestrial carbon-cycling variables under future global change scenarios, and improve the robust projection of global change‒terrestrial carbon feedbacks imposed by Earth System Models.

## Abstract (summary)

**Table:** 

Measurement(s)	organic material • plant production • carbon exchange
Technology Type(s)	digital curation
Factor Type(s)	climate characteristics • vegetation traits
Sample Characteristic - Environment	climate system
Sample Characteristic - Location	global

Machine-accessible metadata file describing the reported data: 10.6084/m9.figshare.12932843

## Background & Summary

As a consequence of fossil fuel combustion and food production, atmospheric CO_2_ concentration and reactive nitrogen deposition have substantially increased^1,2^. Global land temperature has warmed by 0.87 °C over the last 136 years (1880–2015)^3^. However, precipitation trends are more equivocal, with an increase in the northern mid-latitudes whereas trends in other regions cannot be confidently assessed^1^. A consistent manifestation of ongoing climate change is the increasing number of warm days and heavy rainfall events^4,5^. Because plants are subject to regionally different co-limitations by CO_2_, temperature, and the availability of nitrogen and water^6^, global change drivers including elevated CO_2_, atmospheric nitrogen deposition, warming, and changing precipitation regimes can result in complex and likely regionally different effects on ecosystem carbon-cycling variables such as plant production and ecosystem carbon exchange.

Over the last five decades, numerous manipulative experiments have been conducted worldwide and aimed to explore the ecosystem effects of global change drivers. However, the discrepancy and diversity of observations among different ecosystem manipulative experiments have led to a great uncertainty in assessing global change‒carbon cycling feedbacks. Here, we presented a database of 5,213 pairs (the control versus global change treatment) of carbon-cycling variable samples extracted from 2,242 publications that reported results of global change manipulative experiments (GCMEs) over the past four decades (1973–2016). This is an updated version of a previous database (2,496 pairs of samples from 2,230 publications) that was originally used to examine global response patterns of terrestrial ecosystem carbon cycling to single and two combined global change drivers^7^. In the original version, only data collected from the last year when multiple measurements were taken at different years in a study have been used. This updated version releases all year’s data we collected and thus adds an additional 2,717 pairs of samples. The 12 new publications added in this updated version are from the experiments collected in the original version and do not increase the sample size^8–19^.

Besides data from single- and two-factor manipulative experiments, data obtained from the few three- and four-factor experiments are critical to improve the understanding of terrestrial carbon-cycling responses under future global change scenarios. Thus, we also included data extracted from publications that reported three- and four-factor experiments in this updated version. Moreover, the facilities used in CO_2_ enrichment and warming experiments as well as the magnitudes of experimental manipulations were also presented and accompanied with the related experimental data. Furthermore, the references for each pair of samples were added into each dataset to simplify the use and review in the future study. In summary, this database can facilitate the understanding of terrestrial ecosystem carbon cycling to multifactorial global change, and provide empirical data for Earth System Models to reduce the uncertainties in projecting global change‒terrestrial carbon feedbacks.

## Methods

### Publication collection and data compilation

The detailed methods of publication search and data collection were described in our related work^7^. In brief, 10 databases in Web of Science (WoS; 1 January 1900 to 13 December 2016) including BIOSIS Previews, Chinese Science Citation Database, Data Citation Index, Derwent Innovations Index, Inspec, KCI-Korean Journal Database, MEDLINE, Russian Science Citation Index, SciELO Citation Index, and WoS Core Collection were used for searching peer-reviewed publications that reported GCMEs. The 18 keywords for WoS title search were: global change, climate change, free-air carbon dioxide enrichment, free-air CO_2_ enrichment, elevated carbon dioxide, elevated CO_2_, elevated atmospheric CO_2_, CO_2_ enrichment, eCO_2_, [CO_2_], warming, elevated temperature, changing precipitation, increased precipitation, decreased precipitation, nitrogen deposition, nitrogen addition, and nitrogen application. Through these search, 310,177 publication records that might be relevant to our topic were found.

First, we identified all the 310,177 records via reading each title. Second, we read the abstracts of all the records collected in the first step to further screen publications. During the two steps, we excluded 291,436 records because these studies were reviews/meta-analyses or conducted in non-terrestrial ecosystems such as oceans. Third, we read the methods of the remaining 18,741 publications to identify which of them met the following three inclusion criteria:

1. Publications reported results of outdoor GCMEs which had at least three control and global change treatment plots (> = 1 m^2^).
2. The GCMEs were conducted in terrestrial ecosystems except for croplands and lab incubation studies.
3. The GCMEs aimed to examine effects of simulated global change drivers on carbon, nitrogen, and water-cycle variables as well as plant and microbial parameters.

During the screening in the third step, 1,290 publications met these defined criteria.

**Fig. 1:**
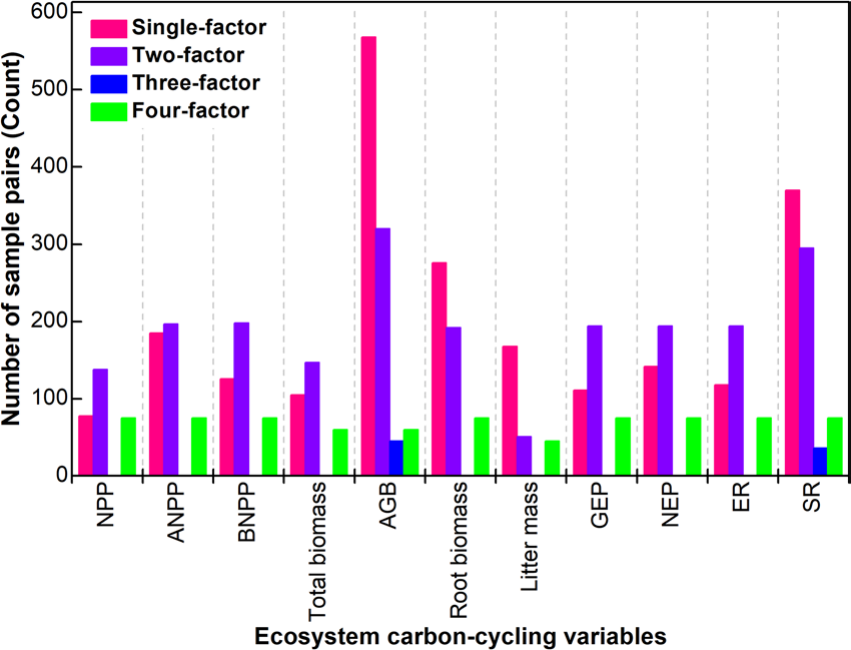
Number of samplings. Number of sample pairs of ecosystem carbon-cycling variables including net primary productivity (NPP), above- and below-ground NPP (ANPP and BNPP), total biomass, aboveground biomass (AGB), root biomass, litter mass, gross and net ecosystem productivity (GEP and NEP), and ecosystem and soil respiration (ER and SR) extracted from publications reporting single-, two-, three-, and four-factor global change manipulative experiments.

We subsequently cross-checked the list of the 1,290 publications with references cited by the previous review/meta-analysis articles in global change research as well as the 1,290 publications, and collected 756 publications. In addition, 184 studies were collected by searching the websites of ecology laboratories and experiment networks and checking the references of the papers downloaded from these websites. In total, 2,230 publications were collected in the original version of the database^7^. Moreover, another 12 publications were found when we checked and reorganized all the data extracted from the 2,230 publications^8–19^. This database compiled 11 plant production and ecosystem carbon exchange variables including net primary productivity (NPP), above- and below-ground NPP (ANPP and BNPP), total biomass, aboveground biomass (AGB), root biomass, litter mass, gross and net ecosystem productivity (GEP and NEP), and ecosystem and soil respiration (ER and SR). Data of mean values, standard deviations or standard errors, and sample sizes (number of plot replications) of these variables in the control and treatment (e.g., elevated CO_2_, nitrogen addition, warming, increased/decreased precipitation, or their combinations) groups were extracted from each publication when possible. The figures were digitized using SigmaScan Pro 5.0 (SPSS, Inc.) and the numerical values were extracted when a publication presented experimental data graphically. Data of the experiments that were conducted over less than one year/growing season were excluded in this database. However, we included short-term data from tundra studies because most of measurements in this ecosystem were performed during July-August. Overall, 5,213 pairs (the control versus global change treatment) of plant production and ecosystem carbon exchange samples were collected in this database, having 2,247, 2,120, 81, and 765 pairs from single-, two-, three-, and four-factor manipulative experiments, respectively (Fig. 1).

### Environmental metadata: Climate and vegetation

Information on the locations and altitudes of each experimental site, site climate including mean annual temperature (MAT) and precipitation (MAP) as well as wetness index $(\frac{{\rm{MAP}}}{{\rm{MAT+10}}})$, ref. ^20^, and vegetation types were extracted from each of the 2,242 publications. If a study did not report climate characteristics for its experimental site, data of MAT and MAP were downloaded from Climate Model Intercomparison Project phase 5 (CMIP5; https://esgf-node.llnl.gov/projects/cmip5/) based on the site coordinate. The dataset selection in CMIP5 was “historical (simulation of recent past 1850–2005)” and the climate data averaged from 20 (i.e. BCC_CSM1_1, BCC_CSM1_1_M, CANESM2, CCSM4, CMCC_CM, CMCC-CMS, CNRM-CM5, CSIRO_MK3_6_0, GFDL_CM3, GISS_E2_H, HADGEM2_AO, HADGEM2_ES, INMCM4, MIROC_ESM, MIROC_ESM_CHEM, MIROC5, MPI_ESM_LR, MPI_ESM_MR, MRI_ESM1, and NORESM1_M)^21^, that contained historical climate data, out of the 35 global climate models available in CMIP5 were used in this study. In addition, we downloaded data of climate means at global 1 × 1° land grid cells from Princeton University (http://hydrology.princeton.edu/data/pgf/v3/) to construct global climate space. Moreover, we classified ecosystems subjected to ecosystem manipulative experiments into five typical types: forests (mature forests and tree seedlings), grasslands (grasslands, meadows, short- and tall-grass prairies, temperate/semi-arid steppes, shrublands, savannas, pastures, and old-fields), tundra, wetlands (peatlands, bogs, marshes, and fens), and deserts.

### Metadata of experimental facilities and performance

Information on CO_2_ enrichment and warming facilities were also extracted from the related publications reporting CO_2_ and warming effects on plant production and ecosystem carbon exchange. Facilities used in elevated CO_2_ experiments included greenhouse, open-top chamber, free-air CO_2_ enrichment, and tunnels. Warming experiments primarily used greenhouse, open-top chamber, soil heating cables, infrared radiator, and infrared reflector to elevate vegetation canopy and soil temperature. In addition, the manipulation magnitudes of global change drivers imposed by manipulative experiments, such as the increases in CO_2_ concentrations (ppm) and temperature (°C), the changes in precipitation amount (mm), and the rates of nitrogen input (g N m^−2^ yr^−1^), were also collected and added into this updated database.

## Data Records

This database was categorized by ecosystem carbon-cycling variables. All datasets were released in figshare^22^. Raw data of ecosystem carbon-cycling variables and auxiliary materials are available as .csv/.txt files including (1) _Abbreviation_.csv, (2) NPP_Dataset.csv (291 pairs of samples), (3) ANPP_Dataset.csv (457 pairs of samples), (4) BNPP_Dataset.csv (399 pairs of samples), (5) Total Biomass_Dataset.csv (312 pairs of samples), (6) AGB_Dataset.csv (993 pairs of samples), (7) Root Biomass_Dataset.csv (543 pairs of samples), (8) Litter Mass_Dataset.csv (264 pairs of samples), (9) GEP_Dataset.csv (380 pairs of samples), (10) NEP_Dataset.csv (411 pairs of samples), (11) ER_Dataset.csv (387 pairs of samples), (12) SR_Dataset.csv (776 pairs of samples), and (13) List of 2,242 publications that reported GCMEs.txt. Each of the 11 datasets contained information on coordinate, altitude, climate, and vegetation type of experimental sites as well as experimental facility and manipulation magnitude of experimental treatments, and sample pairs of data extracted from publications reporting single-, two-, three-, or four-factor GCMEs.

## Technical Validation

The author team made a series of quality assurance and quality control checks for the raw data in the original version^7^ and this updated version. For example, first, several senior authors checked if the extracted data were consistent with the figures or tables in the related publications. Second, whether data extracted from the different studies were from the same experiment were checked. In this updated version, the author team carefully checked all datasets and other related information (e.g., site climate, experimental facility, and manipulation magnitude) again. Given that this updated database will be made publicly available with all data and metadata, further quality checks could be made towards continuing improvement.

**Fig. 2:**
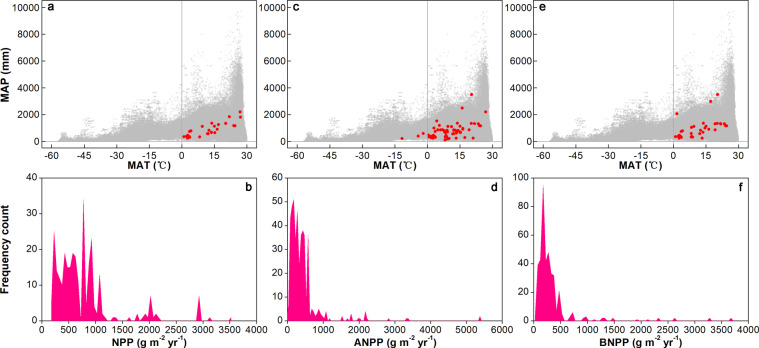
Ranges of local climate and plant productivity. The climate distribution of field experiments with mean annual temperature (MAT) and precipitation (MAP) in the net primary productivity (NPP; **a**), and above- (ANPP; **c**) and below-ground NPP (BNPP; e) datasets (solid red circles). The blank gray circles in panels a, c, and e represent climate means at global 1 × 1° land grid cells (http://hydrology.princeton.edu/data/pgf/v3/). Assessment of frequency distributions of NPP (**b**), ANPP (**d**), and BNPP (**f**) values in the control plots of ecosystem manipulative experiments.

In this updated database, the ranges of NPP, ANPP, and BNPP values (unit: g m^−2^ yr^−1^) are 182–3,519 with a median of 615 (MAT 0.95–27.00 °C and MAP 254–2,213 mm), 30–5,385 with a median of 299 (MAT ‒11.70–26.80 °C and MAP 118–3,550 mm), and 42–3,675 with a median of 224 (MAT 0.95–24.49 °C and MAP 250–3,500 mm), respectively, in the control plots (Fig. 2). By contrast, a previous synthesis has demonstrated that NPP of China’s *Larix* forests is ranging from 422 to 1,621 g m^−2^ yr^−1^ (MAT ‒6.00–6.50 °C and MAP 437–839 mm)^23^. In addition, a global meta-analysis has found that mean NPP are 1,088 ± 111 and 772 ± 93 g m^−2^ yr^−1^ for monoculture plantations and tropical secondary forests, respectively, younger than 18 years old as well as 969 ± 154 and 573 ± 65 g m^−2^ yr^−1^ for monoculture plantations and tropical secondary forests, respectively, 18 years and older^24^. Another work^25^ has showed that NPP of the Canadian Arctic tundra is generally below 1,000 g m^−2^ yr^−1^. Flombaum and Sala (2007)^26^ has estimated ANPP of the Patagonian steppe and yielded a mean ANPP of 57 g m^−2^ yr^−1^. However, a grassland biomass dynamic assessment has revealed that mean grassland ANPP and BNPP worldwide are 331 ± 101 and 745 ± 346 g m^−2^ yr^−1^, respectively^27^.

Given the substantially greater upper limits of plant productivity values in our database than that in several previous works^23–27^, we checked the potential outlier and found that the two NPP values greater than 3,000 g m^−2^ yr^−1^ were extracted from the studies conducted in a temperate forest in Viterbo, Italy (3,106 g m^−2^ yr^−1^)^28^ and a subtropical forest in Southern China (3,519 g m^−2^ yr^−1^)^29^. In terms of ANPP, the four values ranging from 2,209 to 3,383 g m^−2^ yr^−1^ and the two values greater than 5,000 g m^−2^ yr^−1^ were from four northern hardwood forests in Michigan, USA^30^ and a northern forest in Northern Maine, USA^31^, respectively. In addition, the eight BNPP values ranging from 2,325 to 3,675 g m^−2^ yr^−1^ were extracted from a study conducted in a ponderosa pine forest in California, USA^32^. We confirmed that all the anomalously high values of plant productivity were extracted from the related publications correctly.

**Fig. 3:**
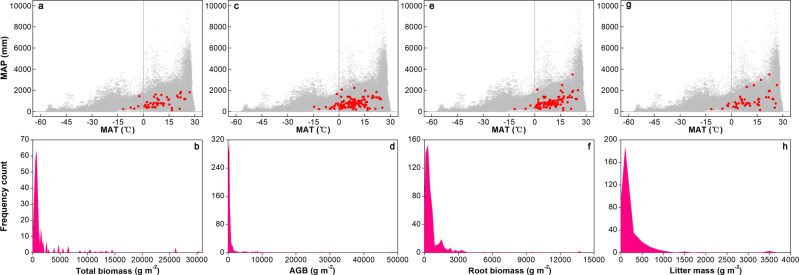
Ranges of local climate and plant biomass. The climate distribution of field experiments with mean annual temperature (MAT) and precipitation (MAP) in the total biomass (**a**), aboveground biomass (AGB; **c**), root biomass (**e**), and litter mass (**g**) datasets (solid red circles). The blank gray circles in panels a, c, e, and g represent climate means at global 1 × 1° land grid cells (http://hydrology.princeton.edu/data/pgf/v3/). Assessment of frequency distributions of total biomass (**b**), AGB (**d**), root biomass (**f**), and litter mass (**h**) values in the control plots of ecosystem manipulative experiments.

Data of plant biomass and litter mass are highly variable (Fig. 3). The medians of total biomass, AGB, root biomass, and litter mass data in the control plots are 698 g m^−2^ (80–30,382 g m^−2^; MAT ‒11.70–27.00 °C and MAP 131–1,845 mm), 308 g m^−2^ (3–47,917 g m^−2^; MAT ‒14.50–25.20 °C and MAP 131–2,218 mm), 388 g m^−2^ (16–13,696 g m^−2^; MAT ‒11.70–25.20 °C and MAP 131–3,500 mm), and 95 g m^−2^ (6–3,579 g m^−2^; MAT ‒11.70–26.50 °C and MAP 131–3,500 mm), respectively. The ranges of total biomass values in our study are comparable to the estimates (0–39,000 g m^−2^) of total biomass of terrestrial biomes in several previous studies^23,33–35^. In addition, several previous AGB assessments^25,35,36^ have found that the ranges of AGB of terrestrial biomes are 0–137,800 g m^−2^. The substantially smaller AGB ranges in our database may be due primarily to the fact that our dataset of AGB has few data obtained from studies conducted in tropical forests^7^. Moreover, a study^37^ estimating forest root biomass has demonstrated that root biomass of terrestrial biomes ranges from 1,181 to 4,651 g m^−2^. Furthermore, a recent plant biomass database has showed that ecosystem-level root biomass and litter mass across all terrestrial biomes are ranging from 0.01 to 1,085 g m^−2^ and from 8 to 224 g m^−2^, respectively^35^.

We further checked and confirmed our data in the datasets of root biomass and litter mass which contained some anomalously high values than that reported in two previous databases^35,37^. We found that the northern hardwood forests in Michigan, USA and a Florida scrub-oak forest on the east coast of Florida, USA had great root biomass densities of 5,318 and 6,522 g m^−2^, respectively^38,39^. In addition, two alpine meadows showed greater root biomass densities (9,524 and 13,696 g m^−2^)^40,41^ than those in forests. In terms of litter mass, two values greater than 3,000 g m^−2^ were obtained from a northern forest in Northern Marine, USA (3,543 g m^−2^)^31^ and an Arctic tundra in Swedish Lapland (3,579 g m^−2^)^42^.

**Fig. 4:**
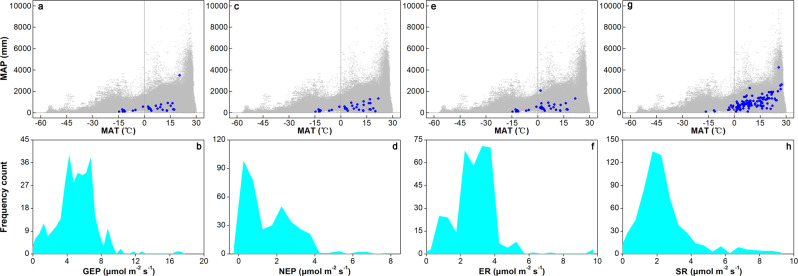
Ranges of local climate and ecosystem carbon exchange. The climate distribution of field experiments with mean annual temperature (MAT) and precipitation (MAP) in the gross (GEP; **a**) and net ecosystem productivity (NEP; **c**), and ecosystem (ER; **e**) and soil respiration (SR; **g**) datasets (solid blue circles). The blank gray circles in panels a, c, e, and g represent climate means at global 1 × 1° land grid cells (http://hydrology.princeton.edu/data/pgf/v3/). Assessment of frequency distributions of GEP (**b**), NEP (**d**), ER (**f**), and SR (**h**) values in the control plots of ecosystem manipulative experiments.

Data of ecosystem carbon exchange are relatively uniform (Fig. 4). The ranges of GEP, NEP, ER, and SR values (µmol m^−2^ s^−1^) are 0.02–17.47 (median = 5.15; MAT ‒14.60–20.30 °C and MAP 101–3,500 mm), ‒0.06–7.67 (median = 1.11; MAT ‒14.60–21.70 °C and MAP 101–1,329 mm), 0.04–9.80 (median = 2.81; MAT ‒14.60–21.70 °C and MAP 101–2,070 mm), and 0.03–9.33 (median = 2.19; MAT ‒16.50–27.40 °C and MAP 101–4,239 mm), respectively, in the control plots. In this study, positive and negative NEP values represent net carbon uptake and loss, respectively.

## Data Availability

Code used in MATLAB 2016b to plot global climate space is available via 10.6084/m9.figshare.7442915^22^.
